# Variation of daily warm season mortality as a function of micro-urban heat islands

**DOI:** 10.1136/jech.2008.078147

**Published:** 2009-07-06

**Authors:** A Smargiassi, M S Goldberg, C Plante, M Fournier, Y Baudouin, T Kosatsky

**Affiliations:** 1 Institut National de Santé Publique du Québec, Quebec, Canada; 2 Département de Santé Environnementale et Santé au Travail, Université de Montréal, Montreal, Quebec, Canada; 3 Centre de Recherche Léa Roback, Université de Montréal, Montreal, Quebec, Canada; 4 Department of Medicine, McGill University, Montreal, Quebec, Canada; 5 Division of Clinical Epidemiology, McGill University Health Centre, Montreal, Quebec, Canada; 6 Direction de Santé Publique de Montréal, Montréal, Québec, Canada; 7 Département de Géographie, Université du Québec à Montréal, Montréal, Québec, Canada

## Abstract

**Background:**

Little attention has been paid to how heat-related health effects vary with the micro-urban variation of outdoor temperatures. This study explored whether people located in micro-urban heat islands are at higher risk of mortality during hot summer days.

**Methods:**

Data used included (1) daily mortality for Montreal (Canada) for June–August 1990–2003, (2) daily mean ambient outdoor temperatures at the local international airport and (3) two thermal surface images (Landsat satellites, infrared wavelengths). A city-wide temperature versus daily mortality function was established on the basis of a case-crossover design; this function was stratified according to the surface temperature at decedents’ place of death.

**Results:**

The risk of death on warm summer days in areas with higher surface temperatures was greater than in areas with lower surface temperatures.

**Conclusions:**

This study suggests that measures aimed at reducing the temperature in micro-urban heat islands (eg, urban greening activities) may reduce the health impact of hot temperatures. Further studies are needed to document the variation of heat-related risks within cities and to evaluate the health benefits of measures aimed at reducing the temperature in micro-urban heat islands.

Epidemiological studies of urban populations have shown that daily mortality increases as ambient temperatures rise above a city-specific threshold.^1^^–^3 Time-series and case-crossover approaches have been used to estimate the association between temperature and daily mortality;^1^ ^4^ ^5^ the strongest effects have been found for temperature on the same or previous day.^1^ ^3^^–^5

Knowledge of the factors that influence the risk of heat-related mortality can be used to develop intervention programmes. In developing such programmes, it is essential to identify particularly vulnerable people and places. As such, the risk of death with high ambient temperatures is higher for people without air conditioning and for those who live on the upper floors of apartment buildings and thus tend to be exposed to higher temperatures indoors.^6^

Ambient temperatures can vary considerably within cities and their surroundings. Spectral bands of satellite images have shown thermal surface distributions that can vary by as much as 10°C.^7^ ^8^ The urban areas in which temperatures are higher are referred to as micro-urban heat islands, and these develop because heat is trapped in large masses that have high specific heats, such as buildings, roadways and parking areas. As well, poor circulation of air in narrow streets and lack of green spaces that provide shade, trap less heat and even dissipate heat through evapotranspiration exacerbate these problems (www.epa.gov/hiri/about/index.html).

Despite the fact that temperatures are not uniform within cities, few health studies of the effects of hot weather have taken such heterogeneity into account. Most studies on the effects of hot weather attribute temperatures to an entire region based on a small number of meteorological stations often located at airports. The objective of the present study was to determine whether people located in micro-urban heat islands, and who are more likely to be exposed to higher temperatures, are at higher risk of mortality during warmer summer days.

## METHODS

### Study population

The study comprised all persons living on the island of Montreal, Canada, during the period 1990–2003. We obtained daily mortality data from the Quebec Institute of Public Health Info-Centre for all such people whose place of death was Montreal. The project was carried out in the context of the Quebec health surveillance plan, which obtained ethics approval from the Quebec Public Health Ethical Health Surveillance Committee. The daily mortality data included individual information on primary and secondary causes of death and personal characteristics, including age and place of death (at home, in long-term care facility or in hospital) and six-character postal code (PC) of the place of death and of usual current residence if death occurred in an institution. Long-term care facilities refer to facilities with that unique function and exclude hospitals that have allocated a certain proportion of their beds to long-term care. The geographical centroid of the PC of the reported place of death was used as the geographical location at the time of death. The geographical centroid of the PC of decedents’ usual residence was used in assigning socioeconomic status. There are more than 50 000 PCs on the island of Montreal, of which about 30 000 represent residential areas; within the urban core, a residential PC corresponds roughly to a road block face within which about 50 individuals live (www.statcan.gc.ca). Large institutions usually have a unique postal code.

The study was confined to the summer months of June, July and August, 1990–2003. We included for analysis all underlying non-accidental causes of death (excluding ICD-9 800–999 and ICD-10 S00–T98), and separately cardiovascular (ICD-9 360–459, ICD-10 I00–I99) and respiratory (ICD-9 460–519, ICD-10 J00–J99) deaths.

### Outdoor ambient temperatures

We used two sources of information for estimating temperature. First, daily mean and maximum outdoor temperatures (from 00:00 to 23:00) were acquired from the Environment Canada Meteorological Centre located at the Pierre Elliott Trudeau International Airport, Dorval, Quebec, Canada, about 20 km from the city core (www.weatheroffice.ec.gc.ca/canada_f.html).

Second, in an attempt to identify persons located in micro-heat islands at the time of death, we attributed a surface temperature to decedents’ reported place of death by positioning each PC geographical centroid on a thermal surface map. Using these centroids, we assigned surface temperatures to deaths that occurred from 1990 to 1996 and from 1997 to 2003 to surface temperature maps from 1990 and 2001, respectively.

The 2001 thermal surface map was obtained by treating an image captured by the Landsat-7/Thematic Mapper (ETM+) satellite (11 August 2001 at 10:25); the 1990 map was obtained from the Landsat-5/ETM+ (11 July 1990 at 10:05). These satellites contain various detectors including one for thermal infrared wavelengths (band 6: 10.4–12.5 μm). At the time the two images were captured, the sky was generally clear; the temperature at the international airport was 23°C for the 2001 image and 21°C for the 1990 image. The Landsat sensors record digital numbers for each pixel which are then converted into surface temperatures using the formulae provided in: http://landsathandbook.gsfc.nasa.gov/handbook/handbook_htmls/chapter11/chapter11.html.

### Ambient levels of air pollutants

Hourly measurements of ozone (O_3_) at fixed-site monitoring stations (on average seven stations) were obtained from the Montreal Environmental Service. Hourly concentrations were averaged over all stations and daily mean and maximum concentrations of O_3_ (from 00:00 to 23:00) were computed from these values.

### Small area indicators of socioeconomic status: the post code dwelling value

We used residence at time of death and linked that to the Montreal property assessment tax database to estimate the socioeconomic status. In population health studies, property assessments can portray socioeconomic status at a smaller scale and with greater geographical flexibility than the commonly used indicators derived from census data.^9^

Specifically, for each PC of residence, we obtained the dollar residential value of the buildings from the property assessment tax database (2001, updated in 2004). This database also contains the number of dwellings found within each residential building. The residential value of the buildings were summed over the PC and then divided by the number of residential units (dwelling) within a PC to obtain an average value of the dwelling.

### Data analyses

We used a case-crossover design in which control days for each death were selected using the time-stratified approach.^10^ ^11^ In this design, the study period was divided into monthly strata and control days for each case were selected as the same day of the week in the month as when they died.

The ambient temperature–mortality relationships were assessed using conditional logistic regression (SAS V.8.02), in which the ambient temperatures (as measured at the airport meteorological station) of several control periods were compared with temperatures during the hazard period, on the day of death and days just before the date of death. To characterise non-linear relationships, we used natural cubic spline functions. Cubic basis functions were constrained to be continuous at the following cut-points (knots): 5th, 33th, 66th and 95th percentiles.^12^ The number of knots was selected based on graphical inspections as well as minimising the Akaike information criterion (AIC). Graphs of such non-linear relationships were created using an SAS macro developed by Heinzl and Kaider^13^ (www.meduniwien.ac.at/msi/biometrie/programme/Rcs.htm). The macro produces odds ratios (ORs) and creates graphs that compare estimates of effect at varying ambient temperatures with the effect estimate at a reference value.

The associations between mortality and temperature derived from measurements at the airport were developed across lag 0 days, lag 1 day (the day before death) and the average of lag 0 to lag 1 days. We also conducted separate analyses among those older than 65 years of age and for those whose primary cause of death was attributed to cardiovascular or respiratory diseases. Separate analyses were conducted including only those who died at home or in long-term care facilities, excluding persons who died in hospital.

To account for the potential confounding effect of air pollution occurring in warm weather, we adjusted for daily mean concentrations of O_3_ evaluated for the same lag period as temperature. We found that response functions for O_3_ were consistent with linearity.

We assessed effect modification of the associations found using ambient temperatures at the airport by surface temperatures at the place of death (using maps of surface temperatures derived from the satellite images). Two strata of surface temperatures were created using several cut-points (50th, 66th and 75th percentiles of the distribution of the surface temperatures at the PCs of places of death). The surface temperature corresponding to a given cut-point was different for each map of surface temperatures and thus years of deaths. For example, the 75th percentile of the 2001 map was 31°C whereas it was 28°C for the 1990 map. Temperature–mortality response functions were then estimated for the “hot” and the “cool” areas for all years of death combined. The associations were derived by considering deaths (and corresponding referent days) within these two areas. Sensitivity analyses were also performed using three strata instead of two (cut-points at the 25th and 75th or at the 33rd and 66th).

To account for the effect of the socioeconomic status of subjects, we further stratified the functions derived from surface temperatures described above with strata based on the residential PC dwelling values. Strata below and above the 25th, 33rd and 50th percentiles of the distribution of the PC dwelling values were created.

## RESULTS

During the summers of 1990 to 2003, 51 689 deaths were recorded in Montreal. Of this number, 3417 deaths were eliminated because of missing PC or missing surface temperature at the place of death. Of the 48 272 deaths (table 1), 14% of deaths occurred at home and 16% of deaths were in long-term care facilities. Most deaths occurred in hospitals. The environmental variables considered in the analyses are also presented in table 1. The mean (SD) ambient temperature during the summers of 1990–2003 varied from 18.6°C (2.4°C) to 21.4°C (3.2°C).

**Table 1:** Distributions of environmental variables and number of deaths, Montreal, June–August 1990–2003

	Number of deaths	Number of days of measurements	Mean (SD)	Minimum	25th percentile	75th percentile	Maximum
Total daily deaths	48 272	1288	31.5 (7.1)	15	33	42	97
Deaths at home	6888		5.3 (2.6)	0	4	7	38
Deaths in hospital	33 523		26.6 (5.3)	8	22	29	54
Deaths in long-term care facilities	7861		6.1 (2.8)	0	4	8	20
Total, ⩾65 years	37 689	1288	29.3 (6.3)	11	25	33	73
Cause of death							
Respiratory	3675	1288	2.9 (1.8)	0	1	4	10
Cardiovascular	16 433	1288	12.8 (4.0)	1	10	15	45
Daily mean ambient temperature (°C)*	–	1288	20.4 (3.2)	9.6	18.2	22.6	29.3
Daily maximum ambient temperature (°C)*	–	1288	24.9 (3.7)	12.2	22. 8	27.2	35.6
Daily mean O_3_ (μg/m^3^)	–	1288	42.3 (20.1)	3.5	28.5	51.4	133.6
Dwelling value (at six-character postal codes of former residence, $C) computed from the tax property assessment)†			56 946 (51 462)	465	28 492	67 400	903 050
Surface temperature at residence of decedents (°C)							
From satellite image taken on 11 July 1990‡			26.4 (2.3)	12	26	28	32
From satellite image taken on 11 August 2001‡			30.2 (1.8)	20	29	31	39

*Ambient temperatures from observations at Pierre Elliott Trudeau International Airport.

†n = 14 861 postal codes of residences.

‡Only one surface map was used for decedents and the surface temperature attributed to a postal code of place of death thus remains the same during control and hazard periods; n = 4790 postal codes at the places of death.

Table 2 presents the associations between the ambient daily mean temperature, as derived from measurements at the airport, for the different exposure lags and causes of death, unadjusted and adjusted for mean concentrations of O_3_. The ORs are expressed as increments of 2°C in mean ambient temperatures. The association between temperature and mortality is curvilinear, with a linear component close to zero, and then increasing gradually until about 20°C, after which the increase in relative risks was exponential. There was little confounding from the effects of daily O_3_. The correlation between the daily mean ambient temperature at lag 0 and daily mean concentrations of O_3_ at lag 0 was 0.46. On days with an average daily ambient temperature of 26°C compared with days with an average of 20°C, the OR for mortality (total non-accidental mortality) was 1.16 (lag 0 days, OR_26°vs20°C_ = 1.04×1.05×1.06; 95% confidence interval (CI) 1.12 to 1.19). ORs of dying at elevated daily ambient temperatures were greater for the 2-day mean (0–1 days) than for lag 0 or lag 1 days alone (table 2).

**Table 2:** Risk of non-accidental mortality for increments of 2°C in daily mean ambient temperature*, evaluated at lag 0, lag 1 and lag 0–1 days, in Montreal, summer 1990–2003

	Unadjusted OR (95% CI)	OR adjusted for O_3_ (95% CI)†
*Total non-accidental mortality*		
Lag 0 days		
20–22°C	1.04 (1.02 to 1.06)	1.05 (1.03 to 1.07)
22–24°C	1.05 (1.04 to 1.07)	1.06 (1.04 to 1.08)
24–26°C‡	1.06 (1.03 to 1.08)	1.07 (1.04 to 1.09)
26–28°C	1.06 (1.03 to 1.08)	1.07 (1.04 to 1.09)
Lag 1 day		
20–22°C	1.02 (1.00 to 1.03)	1.01 (0.99 to 1.03)
22–24°C	1.06 (1.05 to 1.08)	1.06 (1.04 to 1.08)
24–26°C‡	1.09 (1.07 to 1.12)	1.09 (1.06 to 1.11)
26–28°C	1.09 (1.07 to 1.12)	1.09(1.06 to 1.12)
Mean of lag 0–1 days		
20–22°C	1.04 (1.02 to 1.05)	1.04 (1.02 to 1.06)
22–24°C	1.07 (1.05 to 1.08)	1.07 (1.05 to 1.09)
24–26°C‡	1.08 (1.06 to 1.11)	1.08 (1.05 to 1.11)
26–28°C	1.08 (1.06 to 1.11)	1.08 (1.05 to 1.11)
*Respiratory cause of death*		
Lag 0 days		
20–22°C	1.04 (0.98 to 1.10)	1.04 (0.97 to 1.11)
22–24°C	1.08 (1.03 to 1.13)	1.08 (1.02 to 1.14)
24–26°C‡	1.10 (1.02 to 1.20)	1.10 (1.01 to 1.20)
26–28°C	1.11 (1.02 to 1.20)	1.10 (1.01 to 1.20)
Lag 1 day		
20–22°C	1.03 (0.97 to 1.09)	1.02 (0.95 to 1.09)
22–24°C	1.11 (1.06 to 1.16)	1.09 (1.03 to 1.16)
24–26°C‡	1.15 (1.06 to 1.24)	1.13 (1.04 to 1.23)
26–28°C	1.15 (1.06 to 1.25)	1.14 (1.04 to 1.24)
Mean of lag 0–1 days		
20–22°C	1.02 (0.96 to 1.09)	1.01 (0.94 to 1.08)
22–24°C	1.12 (1.06 to 1.18)	1.10 (1.03 to 1.17)
24–26°C‡	1.16 (1.07 to 1.26)	1.14 (1.04 to 1.25)
26–28°C	1.16 (1.07 to 1.27)	1.14 (1.04 to 1.26)
*Cardiovascular cause of death*		
Lag 0 days		
20–22°C	1.03 (1.01 to 1.06)	1.05 (1.01 to 1.08)
22–24°C	1.05 (1.03 to 1.08)	1.07 (1.04 to 1.10)
24–26°C‡	1.06 (1.02 to 1.10)	1.07 (1.03 to 1.12)
26–28°C	1.06 (1.02 to 1.10)	1.07 (1.03 to 1.12)
Lag 1 day		
20–22°C	1.01 (0.98 to 1.04)	1.02 (0.99 to 1.05)
22–24°C	1.07 (1.04 to 1.11)	1.08 (1.05 to 1.11)
24–26°C‡	1.11 (1.06 to 1.15)	1.11 (1.06 to 1.16)
26–28°C	1.11 (1.06 to 1.15)	1.11 (1.07 to 1.16)
Mean of lag 0–1 days		
20–22°C	1.03 (1.01 to 1.06)	1.04 (1.01 to 1.08)
22–24°C	1.07 (1.04 to 1.10)	1.04 (1.05 to 1.12)
24–26°C‡	1.09 (1.04 to 1.13)	1.10 (1.05 to 1.15)
26–28°C	1.09 (1.04 to 1.14)	1.10 (1.05 to 1.15)

*From measurements at Pierre Elliott Trudeau International Airport.

†The same lags were used for temperature and O_3_.

‡26°C represents approximately the 95th percentile of the daily mean ambient temperature at the international airport.

Similar results were found when daily maximum ambient temperatures were used instead of daily means (data not shown). Results for non-accidental mortality among those 65 years or older were similar to those for all ages (data not shown), which was as expected because 78% of deaths occurred in the latter age group. Although ORs for mortality from cardiovascular diseases did not differ from all causes, the risks of dying during hot days of respiratory causes were more pronounced than for non-accidental mortality (lag 0 days, respiratory causes of death OR_26°vs20°C_ 1.24, 95% CI 1.10 to 1.40).

**Figure 1:**
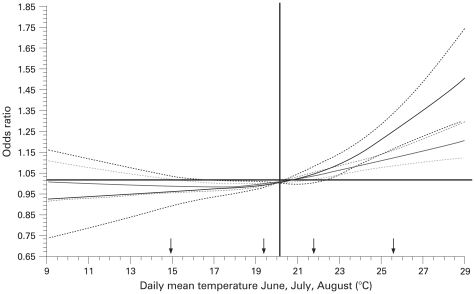
Associations between daily mean ambient temperature, in degrees Celsius, during summer months, 1990–2003, evaluated at lag 0 days and daily non-accidental mortality in Montreal, according to two categories of surface temperatures at the places of deaths (created with satellite images of summer 1990 and 2001). The strata were created with a cut-point at the 75th percentile of the distribution of the surface temperatures at the geographical centroid of the postal codes of the places of death. The black line represents the response function among decedents who were located in “hot areas” at the time of death whereas the grey line is for those who were located in “cooler areas”. Dashed lines represent the 95th confidence limits and are relative to the reference value of 20°C, as indicated by the vertical line. Vertical arrows crossing the *x*-axis locate the four knots used to create the spline functions.

Figure 1 shows on the concurrent day the response function between daily ambient mean temperature and total non-accidental mortality according to strata of surface temperatures at the place of death. The strata were created by dichotomising the distribution of the surface temperatures at the 75th percentile. There were 7588 deaths in the “hot” and 40 684 deaths in the “cool” strata. The proportion of deaths in the “hot” strata was similar when we used only those deaths that occurred “at-home or in long-term care facilities” or “in-hospital”. The slope of the response function was higher for deaths occurring in locations where the surface temperature was higher than in cooler locations; the 95% CIs for the two curves were distinct. The OR comparing mortality on days with a mean temperature of 26°C with that on days at 20°C, at lag 0 days, was 1.28 (95% CI 1.18 to 1.38) in the “hot” strata and 1.13 (95% CI 1.08 to 1.17) in “cooler” strata. Similar response functions were obtained with lag 1 day and lag 0–1 days (data not shown).

The higher risks observed in the “hot” strata are robust to the selection of the cut-point used (50th, 66th, 75th): when the strata were created by dichotomising the distribution of the surface temperatures at the 50th percentile, the OR comparing mortality on days with a mean temperature of 26°C with that on days at 20°C, at lag 0 days, was 1.21 (95% CI 1.15 to 1.27) in the “hot” strata and 1.11 (95% CI 1.06 to 1.16) in “cooler” strata. However, when three categories of surface temperatures were used instead of two, a trend was seen only for the highest category. For example, the OR comparing mortality on days with a mean temperature of 26°C with that on days at 20°C, at lag 0 days, was 1.28 (95% CI 1.18 to 1.38) in the “hot” strata (cut-point >75th), 1.13 (95% CI 1.06 to 1.21) in the “cool” strata (cut-point <25th) and 1.13 (95% CI 1.08 to 1.19) in the “mid” strata.

Subanalyses were performed considering only those who died at home or in long-term care facilities. The effect for deaths that occurred at home or in long-term care facilities was greater than for deaths at all locations (OR comparing total mortality at home or in long-term care facilities on days with a mean temperature of 26°C with that on days at 20°C, at lag 0 days was 1.30, 95% CI 1.23 to 1.38; for deaths at all locations, the similar figure was 1.16, 95% CI 1.12 to 1.19). Analyses performed considering those who died at home or in long-term care facilities also suggest that persons living in micro-urban heat islands and dying at home may be at higher risk than those living in cooler areas during extreme heat events. The ORs for total non-accidental mortality on days with 26°C as compared with days with 20°C, at lag 0 days, among those who died at home and lived in a “hot” area (where the surface temperature strata were created by dichotomising the distribution of the surface temperatures at the 75th percentile), was 1.39 (95% CI 1.23 to 1.58) as compared with 1.28 (95% CI 1.20 to 1.36) among those living in an area where the surface temperature was “cooler”. However, because only 31% of persons died at home or in long-term care facilities, the variability in the estimates was much larger than for the analyses performed for the entire population, even if we expect better classification of exposure for this group than for those who died in hospital, as the duration of stay in the hospital is unknown. We obtain relations similar to those with the entire population when we used only those who died in hospitals (data not shown).

**Figure 2:**
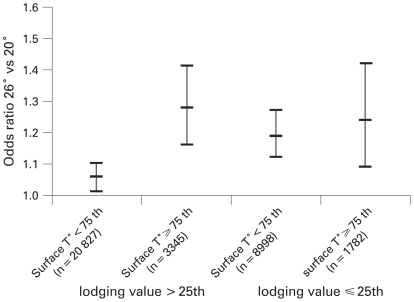
Risk of dying at mean daily ambient temperature of 20°C (lag 0) compared with 20°C, in Montreal during summer 1990–2003, for categories of residential dwelling values (proxy for the socioeconomic status) and categories of surface temperatures at place of death (from a satellite image of summer 1990 or 2001). Deaths that occurred in long-term care facilities were excluded owing to missing former residential dwelling values. There were 5459 deaths with missing dwelling values.

The risk functions presented in fig 1 were further stratified by dwelling values at the PC of usual residence, as derived from property values on the municipal housing tax file. The correlation between the PC dwelling values at the former residences and the PC surface temperatures at the places of death was moderate (r = −0.25). ORs at 26°C relative to 20°C are presented in fig 2 for strata of residential dwelling values and categories of surface temperatures at the places of death. The lowest risk group included those located in “cooler” areas and whose former residence was located within a PC where dwellings had higher dollar values; those dying in “hot spots” and who lived in areas where dwellings had higher values were at higher risk of dying at a daily ambient temperature of 26°C than at 20°C, than those who lived in “cooler” areas where dwelling values were higher. Although risks were higher for those whose former residence was located within a PC where dwellings had low dollar values compared with those in high dollar values, the trend with surface temperature was not as pronounced in areas of low dwelling values. Similar associations were also seen when stratifying based on indicators of socioeconomic status derived from census data instead of property values (data not shown).

## DISCUSSION

Over 35 years ago, Clarke^14^ reported that, during a heat episode in 1966 in St. Louis, Missouri, excess deaths were much more pronounced in the city than outside the city limits and associated this finding with the urban heat island effect. Few studies since have investigated how heat-related health risks vary geographically. Our results suggest that heat-related mortality varies with the surface temperature at the place of death, as derived from satellite imagery.

Surface temperatures derived from satellite imagery present an interesting approach to estimating the intra-urban variation of population heat exposure as very high correlations between surface temperatures and ambient temperatures have been reported.^15^ We also showed that indoor dwelling temperatures vary both with surface temperatures derived from infrared imagery and with ambient temperatures, as they complement each other to represent exposure.^16^ However, further studies are needed to document the accuracy of infrared imagery to represent population exposure as high correlations have not always been reported between surface and ambient temperatures. Correlations with air temperatures have been reported to depend strongly on factors such as the location of the air temperature measurement sites, the land surface type and climate characteristics.^17^

Intra-urban variation of heat-related deaths was suggested by a recent case–control study showing that the surface temperature derived from satellite imagery around the residence of those who died was an important risk factor during the August 2003 heat-wave in France.^18^ Smoyer^19^ also reported higher mortality rates during severe heat waves in denser, potentially warmer and also more deprived areas of St. Louis, Missouri.

We made use of “population-averaged” temperatures and did not have access to individual data that would allow us to classify individuals by actual levels of temperature. In particular, all subjects were assumed to be at the location of their death (ie, home, hospital, long-term care facility) during the hours before their death, but we did not have information as to their real location on “case” and “control” days. We also did not have information as to which floor of a building they were located on or whether they used an air conditioning system. While misclassification of exposure is likely in our study, the extent of the errors would not be affected by air conditioning as most deaths in Montreal occurred in hospitals and there is little use of air conditioning systems in these hospitals (Régie Régionale de la Santé et des Services Sociaux de Montréal, personal communication, 2009).

When analyses were subdivided into those whose residences were located in areas with dwellings of high or low values, a trend with surface temperature was mostly seen in areas with dwellings of high values. This finding suggests that the health of those who lived in areas of low dwelling values and probably of lower socioeconomic status may be influenced by other risk factors more strongly than by the surface temperature at their place of death.

A limitation of this paper is that we used only two maps of surface temperatures for mortality data that spanned from 1990 to 2003. As the urban structure has changed throughout the years, misclassification of micro-urban heat islands will also have changed. Although stratification of death by surface temperature using only the satellite image from 1990 yielded similar results to those using only the 2001 image (data not shown) or to those using both images (fig 1), using maps for each summer would reduce misclassification of the micro-urban heat islands.

As with others,^4^ ^5^ ^20^^–^22 we found a non-linear association between mean daily ambient temperature and daily mortality, with a short latency for the effect of high temperatures on mortality. Interestingly, the effect on at-home mortality was greater than for deaths in hospital, perhaps reflecting the suddenness of heat-related deaths. There was little confounding from the effects of daily O_3_ levels. As both O_3_ and ambient temperatures vary together in time some have suggested that O_3_ and other air pollutants may confound the temperature–mortality relationship,^23^ ^24^ but we observed little confounding.

The results of our study suggest that in urban areas where exposure to temperature was higher, the risk of death was greater during the warmest summer days. However, further studies are needed to document that heat-related risks vary within cities and to document how measures aimed at reducing the temperature of the micro-urban heat islands (eg, urban greening activities) may complement interventions at the individual level (eg, the use of air conditioning).

**What is already known on this subject**

Epidemiological studies of urban populations have shown that daily mortality increases as ambient temperatures rise above a city-specific threshold. Despite the fact that ambient temperatures are not uniform within cities, in few health studies of the effects of hot weather has such heterogeneity been taken into account.

**What this study adds**

Our results suggest that people located in micro-urban heat islands, and who are more likely to be exposed to higher temperatures, may be at higher risk of mortality during warm summer days.
